# Realising precision oncology through shared real-world data infrastructure

**DOI:** 10.2340/1651-226X.2026.45074

**Published:** 2026-02-22

**Authors:** Andreas Bjerrum, Andreas Fanø, Ulrik Lassen

**Affiliations:** a Department of Oncology, Rigshospitalet, Denmark; b Roche Pharmaceutical A/S, Copenhagen, Denmark

**Keywords:** Precision medicine, secondary data analysis, costs and cost analysis, health data governance, public-private partnership, real-world data, outcomes-based pricing negotiations

## Oncology in transition

Oncology is in a major transition [1]. The development of targeted therapies has not only expanded treatment options but also introduced new evidence challenges. Precision oncology, characterised by molecularly defined targets and small patient subgroups, has seen a rise in conditional approvals grounded in biological activity and unmet need. Some of these drug approvals rely on small, single-arm basket trials, because the traditional comparative effectiveness approaches are often infeasible due to limited trial feasibility or lack of equipoise [2, 3].

As a result, once new medicines enter clinical practice, uncertainty persists about how they should be positioned within treatment pathways and which patients derive the most benefit. Healthcare systems face increasing pressure, driven by ageing populations, workforce shortages and rising cancer incidence and higher cancer prevalence from improved survival. These factors have placed high demands on health systems [4], amid growing expectations for financial sustainability.

There is a need to generate evidence that not only meets regulatory requirements for benefit–risk assessments but also meets health technology assessment (HTA) requirements for cost-effectiveness and reimbursement decisions. In this context, the secondary use of health data – that is, data generated in routine care but used for purposes beyond the individual patient’s treatment – has become critical.

## Shift in evidence demands

The pharmaceutical industry, as marketing authorisation holder, is responsible for generating the clinical evidence needed to bring new medicines to market and maintain authorisation. However, with regulatory agencies increasingly relying on conditional approvals, this trend has shifted part of the evidence-generation burden into the post-marketing phase, where secondary use of real-world data (RWD) supports the efforts to address the limitations of clinical trial evidence [2, 5, 6].

This redistribution of *evidence responsibility* has major implications. Although electronic health records (EHRs) systematically capture clinical data, they were built for documentation and administration, not evidence generation. Transforming data into research-ready datasets is complex requiring integration of clinical and data science expertise to ensure data accuracy, completeness and traceability [7]. Moreover, data most critical for oncology, including therapeutic responses, treatment toxicities and disease progression, are predominantly unstructured and therefore challenging to capture, curate and interpret.

This raises a fairness question: if post-approval data collection forms part of the evidence base required for the continued market access of commercial products, should the financial and operational responsibility rest with the public sector? We argue that it should not. Private stakeholders should help fund and maintain RWD infrastructure, ensuring more equitable sharing of the burden of evidence production; in return, it should have access to analyses that align with the industry’ evidence needs. For example, the pharmaceutical industry increasingly requires a comprehensive understanding of the evolving biomarker landscape across tumour types and patient populations.

## Evidence paradigm in real-world precision oncology

Many potential druggable targets are known, some with therapies approaching regulatory approval and others still in early development. Yet, routine testing for emerging biomarkers is inconsistently implemented and therefore rarely captured in RWD, limiting the ability to evaluate biomarker prevalence, treatment patterns and outcomes at scale.

The master trial concept is traditionally defined as an overarching interventional master protocol designed to evaluate multiple therapeutic hypotheses, such as different treatments, disease subtypes or biomarker-defined populations, within a single coordinated clinical trial framework [8–10] or coordinated trial networks [11]. These designs aim to improve efficiency in drug development through shared infrastructure and adaptive evaluation of experimental interventions.

Master observational trials (MOTs) extend this paradigm to a non-interventional, real-world evidence setting that encompasses the entire patient population, including individuals receiving standard-of-care therapies. Rather than assigning treatments, MOTs combine protocol-driven observational rigor with the breadth of RWD to systematically capture molecular diagnostics, therapeutic exposures and outcomes across standard-of-care populations [12]. While informed by the operational principles of master protocols, MOTs differ fundamentally in design and intent, enabling comprehensive biomarker evaluation across the full patient population without experimental intervention.

Copenhagen Master Observational Trial (C-MOT) is part of the Danish OSCAR (One-Stop Shop for Clinical Research) initiative, which exemplifies how public–private collaboration can strengthen data collection, integration and evidence generation [13]. OSCAR is a partnership between healthcare authorities, government, industry stakeholders and patient organisations that aims to improve access to and utilisation of healthcare data. By combining the registry-based RWD, EHR data and whole-genome sequencing (WGS) within a secure governance framework, OSCAR demonstrates shared public–private responsibility for RWD generation. While registries, EHRs and WGS are largely produced within the public healthcare system, sustained private-sector research and innovation are essential to enable scalable, secure and interoperable data infrastructures used in the project.

Looking ahead, the ambition for C-MOT is to evolve into a population-level platform covering all patients in participating clinics. In this model, comprehensive molecular profiling would be embedded into standard clinical pathways and linked with clinical data capture. This could support continuous learning, accelerating the identification, validation and uptake of emerging biomarkers and targeted therapies.

Realising this vision, however, depends on addressing the structural and governance challenges inherent to public–private partnerships. As post-marketing RWD increasingly underpin regulatory and scientific decision-making, the development of research-grade data infrastructures requires sustained investment and time. In the absence of clear alignment on responsibilities, timelines and funding, private-sector involvement may remain transient, potentially undermining the long-term viability of such initiatives.

## Building sustainable data ecosystems

Biomarker advances have divided common cancers into rare molecular subtypes, leaving single-site cohorts too small for robust analysis and highlighting the need for multi-site, cross-border data networks [14, 15]. While Denmark and the other Nordic countries maintain some of the most advanced RWD sources, effective linkage for such ‘rare cancers’ depends on sustained investment in data standardisation, interoperability and analytic capability.

Adopting shared frameworks such as the Observational Medical Outcomes Partnership Common Data Model (OMOP CDM) can facilitate this process [16]. OMOP enables consistent variable definitions across projects and supports federated research models, where data remain local but analytic scripts and aggregated results are exchanged. This approach aligns with GDPR and the forthcoming European Health Data Space (EHDS) regulation, which mandates secure access through authorised data bodies [17].

The OMOP framework supports quality, compliance and multinational research collaborations such as VALO (Value from Nordic Health Data) and FALCON-Lung [18, 19]. These projects use federated analyses to benchmark care quality and treatment outcomes across Nordic countries, illustrating the scalability of OMOP CDM.

## Towards a learning oncology system

The increasing use of RWD is a major development in modern oncology. Evidence generation, once largely industry led, is now a shared societal task. As approvals increasingly depend on data generated in the public health system, the sustainable progress requires co-funded, co-governed partnerships aligning commercial interests and public priorities.

Projects such as OSCAR, C-MOT and VALO represent efforts to explore such models in practice. While still evolving, these projects underscore key considerations, including fairness in cost-sharing, transparency in governance, harmonised data standards and the need for sustained investment in technological and analytical capacity. By embracing these principles, the Nordic countries can lead the global transition towards a learning oncology system – one in which evidence generation is embedded in everyday care, ensuring that data from every patient’s care translates into faster, safer access to new treatments.

## Data Availability

NA.
